# Costs and consequences of abortions to women and their households: a cross-sectional study in Ouagadougou, Burkina Faso

**DOI:** 10.1093/heapol/czu025

**Published:** 2014-05-14

**Authors:** Patrick G C Ilboudo, Giulia Greco, Johanne Sundby, Gaute Torsvik

**Affiliations:** ^1^Département de Santé Publique, Unité de Recherche sur les Politiques et Systèmes de Santé, Centre Muraz, 2054 Avenue Mamadou Konaté, 01 BP 390 Bobo-Dioulasso, Burkina Faso ^2^Agence de Formation, de Recherche et d’Expertise en Santé pour l’Afrique (AFRICSanté), 773 Rue Guillaume Ouédraogo 01 BP 298 Bobo-Dioulasso, Burkina Faso ^3^Department of Community Medicine, University of Oslo, Post Box 1130 Blindern, 0317 Oslo, Norway ^4^Health Economics and Systems Analysis Group, Department of Global Health and Development, London School of Hygiene and Tropical Medicine 15-17 Tavistock Place, London, WC1H 9SH, United Kingdom ^5^University of Bergen and Chr Michelsen Institute, P.O.Box 6033 Bedriftssenteret, N-5892 Bergen, Norway

**Keywords:** Abortions, costs, consequences, Ouagadougou, Burkina Faso

## Abstract

Little is known about the costs and consequences of abortions to women and their households. Our aim was to study both costs and consequences of induced and spontaneous abortions and complications. We carried out a cross-sectional study between February and September 2012 in Ouagadougou, the capital city of Burkina Faso. Quantitative data of 305 women whose pregnancy ended with either an induced or a spontaneous abortion were prospectively collected on sociodemographic, asset ownership, medical and health expenditures including pre-referral costs following the patient’s perspective. Descriptive analysis and regression analysis of costs were performed. We found that women with induced abortion were often single or never married, younger, more educated and had earlier pregnancies than women with spontaneous abortion. They also tended to be more often under parents’ guardianship compared with women with spontaneous abortion. Women with induced abortion paid much more money to obtain abortion and treatment of the resulting complications compared with women with spontaneous abortion: US$89 (44 252 CFA ie franc of the African Financial Community) vs US$56 (27 668 CFA). The results also suggested that payments associated with induced abortion were catastrophic as they consumed 15% of the gross domestic product per capita. Additionally, 11–16% of total households appeared to have resorted to coping strategies in order to face costs. Both induced and spontaneous abortions may incur high expenses with short-term economic repercussions on households’ poverty. Actions are needed in order to reduce the financial burden of abortion costs and promote an effective use of contraceptives.

**KEY MESSAGES**

- Costs of treating induced and spontaneous abortions and their complications consume significant resources of women and their households in Ouagadougou.
- Women continue to pay much more money than what was recommended by the national policy of normal and emergency deliveries for accessing postabortion care.
- There is a need for monitoring the implementation of postabortion care subsidy to ensure that the policy is rigourously applied and that the targeting mechanism effectively reaches the people who need it.

## Introduction

The Millennium Development Goals highlighted the need to accelerate the reduction of maternal mortality and morbidity (UN 2013). Consequently, various policy interventions such as safe motherhood initiatives have been implemented worldwide (Starrs 2006). Maternal mortality ratio reductions have been reported around the world (Hogan *et al.* 2010). In spite of this, each year, hundred thousands of women continue to die throughout the world (WHO 2012b). A larger part of these deaths (66 500) is attributable to induced abortion (Rasch 2011). In addition to these deaths, which could often have been prevented with appropriate care (Berer 2004; WHO 2012a), millions of other women suffer from various abortion related complications (WHO 2012a).

There are several studies that have examined the incidence of abortion and abortion-related complications treated in hospitals (Rossier *et al.* 2006; Singh 2006; Åhman and Shah 2011; Sedgh *et al.* 2011, 2012; Shah and Åhman 2012). Several other studies have also documented access to postabortion care and the economic consequences of induced abortion (Johnston *et al.* 2007; Henshaw *et al.* 2008; Vlassoff *et al.* 2009a, 2012; Shearer *et al.* 2010; Singh 2010; Singh *et al.* 2012). Most of the studies that reported the economic consequences of induced abortion focused on estimating the financial burden of the treatment of its complications. They demonstrated that treatments of induced abortion complications may consume large proportions of health system resources (Johnston *et al.* 2007; Henshaw *et al.* 2008; Shearer *et al.* 2010; Babigumira *et al.* 2011; Vlassoff *et al.* 2009b, 2012). However, studies that report the costs associated with induced and spontaneous abortions to women and their households are scarce. Yet costs of abortions, particularly those of induced abortions, may be of high importance. Such costs may deplete many households of resources, thus reducing their ability to afford other healthcare services.

In places where abortion is restricted by law or by social norms, studies have shown that women often have to resort to the informal health sector in order to end their unwanted pregnancies (Okonofua 2006; Benson *et al.* 2011). The conditions under which these abortions are performed often lead to complications (Banerjee and Andersen 2012), which will, in most cases, require hospital care (Singh 2006). Postabortion care for treating complications of abortions in hospitals may come in addition to amounts paid for terminating the pregnancy and lead to higher expenses for women who have had an induced abortion compared with women with a spontaneous abortion or normal birth.

Furthermore, women with induced abortions may be reluctant to truthfully report their abortions due to stigma, shame and fear of prosecution. Reluctance to report abortions may lead to misclassification biases (Singh 2006; Bernabé-Ortiz *et al.* 2009). Additionally, it may cause women with induced abortion to delay their access to postabortion care. The delay in requesting postabortion care may lead to more severe complications which, in turn, may incur much higher expenses, especially for women from low socio-economic status who may have been forced to resort to cheaper but more dangerous abortion methods. Because of that, it is hypothesized that some women, particularly those who have had induced abortions, may have faced unaffordable or ‘catastrophic’ costs.

In 2006, the Government of Burkina Faso, a West-African country that faces a weak health system (Ridde *et al.* 2011), a low rate of contraceptive use (Vlassoff *et al.* 2011) and a restrictive abortion law (Guttmacher Institute 2014), implemented a subsidy policy for delivery and emergency obstetric care which, among other efforts, subsidizes 80% of postabortion care costs (Ministère de la santé Burkina Faso 2006). The remaining 20% (3600 CFA), broadly equivalent to US$7, is charged to the woman. Although the primary reason for this policy was to make postabortion care affordable by reducing costs of care, no study has been conducted to assess the household costs, both direct and indirect (including complications costs), associated with spontaneous and induced abortions. Additionally, relatively few studies have addressed the economic consequences that these payments may have for women and their households. In Burkina Faso, both legal and illegal, safe and unsafe abortions are prevalent. Therefore, it is critical to estimate both the costs and the consequences of abortions for women and their households.

## Methods

### Study type and participants

A cross-sectional study that collected cost data from the patient perspective was conducted between February 2012 and September 2012 in Ouagadougou, Burkina Faso, a West African country in which 46% of the population live under the poverty line (Ministère de la santé Burkina Faso 2010). A total of 307 women with either a spontaneous or an induced abortion were prospectively recruited from two health facilities in Ouagadougou.

The health facilities were selected to guarantee the recruitment of a sufficient number of women with abortion. One health facility was the national referral teaching hospital and the other one was a private clinic with expertise in treating abortion complications.

In each facility, one of the experienced health staff, generally the midwife responsible for the maternity or manual vacuum aspiration ward identified women with a spontaneous or induced abortion based on clinical definitions of abortions. A spontaneous abortion was defined as the loss of a pregnancy without outside intervention before 20 weeks’ gestation (Griebel *et al.* 2005), whereas an induced abortion was defined as a procedure for terminating an unintended pregnancy carried out either by persons lacking the necessary skills or in an environment that does not conform to minimal medical standards, or both (WHO 2011). Additionally, information on the nature of the abortion was also obtained by interviewing women. Abortions were subsequently classified as induced when the clinical ascertainment was confirmed by the woman herself reporting that she had had an induced abortion. All other abortions were classified as spontaneous. This procedure of classifying the cases may have led to some induced abortions being inaccurately classified as spontaneous. Because of this issue, we categorized the groups of women into ‘certainly induced abortion’ and ‘reportedly spontaneous abortion’, with induced abortion and spontaneous abortion referring respectively to these groups.

### Procedure and data collection tools

Once identified, all the women were directed to two female interviewers who were in charge of establishing contact with them for further investigation. All women who met the eligibility criteria were invited to participate in the study. Out of a total of 307 women, 305 accepted to participate in the study, giving a participation rate of 99%. At discharge, subjects who consented to participate in the study were interviewed at the health facility or clinic or at home. The two qualified female interviewers collected data from all the women who had had an induced or a spontaneous abortion using an interviewer administered pilot-tested questionnaire. Prior to fieldwork, the interviewers were given comprehensive training on data collection procedures and on the extraction of selected clinical data from medical records.

Data were collected using two structured pilot-tested questionnaires. The main questionnaire contained questions on a range of socio-economic background information, health, health expenditures (on drugs, ultrasounds, laboratory tests, hospitalization, transport, etc.), as well as pre-referral costs, including costs of drugs, ultrasounds, laboratory tests, hospitalization and transportation. This questionnaire was complemented by a short health worker questionnaire which was intended to extract selected medical information, including the type of postabortion complications, from hospital records. These complications included haemorrhage, infections, injuries to genital organs and incomplete abortions, etc.

### Statistical analysis

Data were double-entered and validated on Epidata Version 3.5. The database was re-coded and transformed into a STATA dataset. Analysis was done in STATA, version 11.2. We conducted a descriptive analysis in order to understand the characteristics and assess the differences between the groups of women. The differences between proportions were tested using the chi-squared test. We resorted to the Mann–Whitney test to test for differences where distributions were skewed. Total cost associated with induced abortion was estimated as the sum of incurred costs for the abortion procedure, including expenses for immediate care before hospitalization, plus costs borne for treating abortion complications in hospitals. Total cost of spontaneous abortion was estimated as the sum of treatment-related expenses before and during hospitalization. In both cases, all total costs included expenses for medicines, laboratory tests, ultrasounds, hospitalization, transportation, etc. To assess households’ socio-economic status, we constructed an asset index based on a multiple component analysis of asset variables such as possession of a radio, television, fridge, bicycle, motorcycle, car, cart; and on housing characteristics such as type of toilets, nature of roof and walls, possession of electricity and type of water supply. We divided the sample into tertiles based on the asset index score, estimated the average cost by socio-economic status and tested differences in mean costs between the groups. To assess unaffordability of abortion costs, we used the approach that defined an unaffordable health care cost as a payment which equals or exceeds 10% of the household income (Garner *et al.* 2004; Russell 2004). However, we did not collect household income because it would not have been possible to collect income at household level without exposing women with secret induced abortion. Alternatively, we used the World Bank estimate of gross domestic product (GDP) per capita as a proxy of annual per capita income to estimate costs as a proportion of income. In 2012, GDP per capita was estimated at US$634 (The World Bank 2014). After adjusting for inflation, it amounts in real value to US$593, i.e. 294 721 CFA.

In a poor country such as Burkina Faso, even a small expenditure on care may force worse off households or individuals to reduce consumption of essential food and other goods, deplete entire savings, borrow with high interest rates, and/or sell assets, etc., contributing therefore to impoverishment (Russell 2004). Because of this, we also considered the coping strategies used in the analysis of unaffordability of abortion costs and presented the proportions of households that resorted to coping strategies to face costs by type of abortion, and tested for significant differences between proportions using the chi-squared test.

Because the health structures in which we recruited women may not necessarily be comparable, we tested for significant differences in total costs of abortion between hospitals by running a linear regression of the mean abortion cost, adjusting for hospital. Half of the women reported partial or incomplete transport costs because the husband/partner who paid/borne this cost item was not present during the interview. This was more often the case for women who had had an induced abortion. Furthermore, many women failed to accurately report opportunity costs (productive days lost) associated with the hospitalization during the interviews. Adding up these costs would have biased our estimates. We therefore excluded both transport and opportunity costs from the analysis. All monetary values are in the Burkina Faso currency CFA (1 US$ = 497 CFA in 2012).

## Ethical consideration

This study was approved by the Ethical Committee of the Ministry of Health of Burkina Faso and the Norwegian Regional Committee for Medical and Health Research Ethics. Written informed consent was obtained from all participants before participation in interviews.

## Results

### Characteristics of the study participants

Table 1 summarizes the socio-demographic characteristics of the respondents. All the women except two agreed to participate in the study. Sixty-one per cent of the women were married. As expected, women with induced abortion (63%) were more often single compared with women with spontaneous abortion (28%; *P* < 0.001). They were also significantly younger than women with spontaneous abortion (*P* < 0.001) and had attended secondary school (*P* = 0.004). Furthermore, a higher proportion of women with induced abortion (71%), compared with women with spontaneous abortion (30%), have had no living children (*P* < 0.001), and were experiencing their first pregnancy (63%) against (24%), respectively. Findings also suggest a higher proportion of women who had an induced abortion (79%), compared with women with spontaneous abortion (15%), were under parents’ guardianship (*P* < 0.001). Finally, a higher proportion of women who had an induced abortion (66%), compared with women with spontaneous abortion (40%), had to undergo manual vacuum aspiration for uterine evacuation.

**Table 1:** Characteristics of the study participants by type of abortion

	All		Certainly induced abortion		Reportedly spontaneous abortion		*P*-value
	*n*	%	*n*	%	*n*	%	
Participation							
Consented	305	99	38	100	267	99	
Refused	2	1	0	0	2	1	NC
Residence							
Allotted area	230	75	32	84	198	74	
Unallotted area	75	25	6	16	69	26	0.23
Education							
Not educated	99	33	4	10	95	36	
Primary education	65	21	8	21	57	21	
Secondary school	117	38	23	61	94	35	
University level	24	8	3	8	21	8	0.004
Marital status							
Married	186	61	2	5	184	69	
Widowed/divorced	21	7	12	32	9	3	
Single/never married	98	32	24	63	74	28	<0.001
Age							
<20	39	13	14	37	25	9	
20–24	83	27	11	29	72	27	
25–29	70	23	9	24	61	23	
30–34	63	21	3	8	60	22	
≥35	50	16	1	2	49	19	<0.001
Number of pregnancies							
1	89	29	24	63	65	24	
2–4	168	55	12	32	156	59	
≥5	48	16	2	5	46	17	<0.001
Number of living children							
0	108	35	27	71	81	30	
1–3	164	54	9	24	155	58	
≥4	33	11	2	5	31	12	<0.001
Chief of the household							
Woman herself	234	77	8	21	226	85	
Woman’s parents	71	23	30	79	41	15	<0.001
Type of uterine evacuation							
Manual vacuum aspiration	133	44	25	66	108	40	
Oral product (misoprostol)	101	33	8	21	93	35	
Missings	71	23	5	13	66	25	0.01

NC, not calculated.

### Costs associated with induced vs spontaneous abortion

Women who had had an induced abortion paid significantly more for the abortion procedure and treatment of its complications than women with spontaneous abortion: US$89 (44 252 CFA) vs US$56 (27 668 CFA), respectively (*P* < 0.001) (Table 2). They paid one and a half times the amount paid by women with spontaneous abortion for ending their pregnancy, and on immediate treatment linked to the abortion procedure before hospitalization: US$56 (28 065 CFA) vs US$37 (18 413 CFA), respectively. Findings also showed that women with induced abortion paid more than one and a half times the amount paid by women who had had a spontaneous abortion for treating complications resulting from their abortions: US$33 (16 187 CFA) vs US$19 (9255 CFA), respectively (*P* < 0.01). Furthermore, there was no evidence of a significant difference between induced and spontaneous abortion costs by type of facility (results not presented).

**Table 2:** Average cost (in US$) associated with induced vs spontaneous abortion to women and their households

	Certainly induced	Reportedly spontaneous	*P*-value
Expenditure before hospitalisation			
Cost of the procedure	56 (63)	—	NA
Cost of care	—	37 (44)	NA
Expenditure during hospitalisation			
Cost of treatment of complications	33 (50)	19 (27)	<0.001
Total cost	89 (75)	56 (50)	<0.01

Standard deviation is given in brackets. NA, not available.

### Costs associated with induced vs spontaneous abortion by socio-economic status (SES)

The distribution of the mean costs to women who had an induced abortion and women with a spontaneous abortion is shown in Table 3. Women from low income households paid the highest amount of money for the abortion procedure and for subsequent treatment of its complications: US$105 (52 231 CFA). For women who had a spontaneous abortion, we also found evidence of differences in the average expenditure on care relative to their socio-economic status (*P* = 0.047). Additionally, in this group, women from low income households also paid the highest amount of money for postabortion care: US$70 (34 765 CFA).

**Table 3:** Average cost (in US$) associated with induced vs spontaneous abortion by socio-economic status

		Mean cost associated with abortions (SD)	
Wealth tertiles	Mean asset index score (respondents)	Certainly induced	Reportedly spontaneous
Low	−1.02 (103)	105 (96)	70 (66)
Middle	−0.19 (102)	86 (46)	49 (42)
High	1.25 (100)	69 (70)	48 (34)
*P*-value		0.37^a^	0.047^a^

^a^Linear regression of log-transformed abortion and postabortion care cost on wealth tertiles. SD = Standard Deviation.

**Figure 1:**
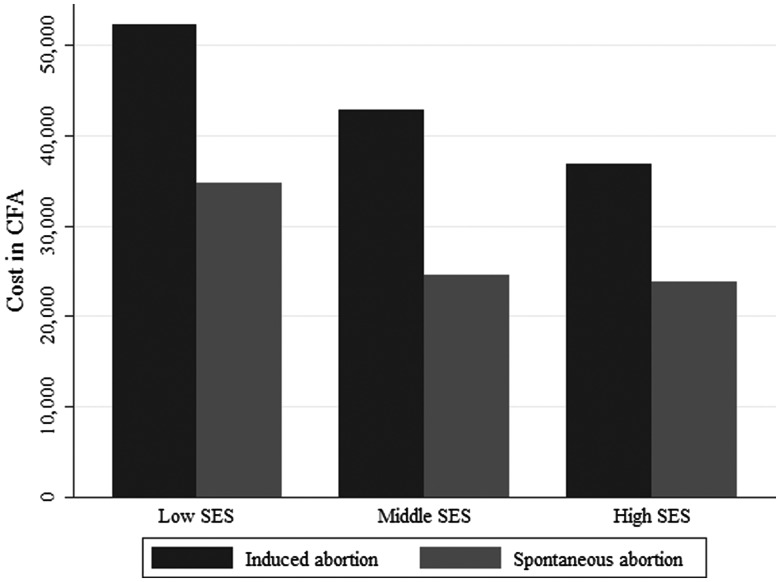
Average cost (in CFA) of induced and spontaneous abortion by socioeconomic status of households.

Furthermore, whatever the socio-economic status of the household, women who had an induced abortion paid much more money compared with women who had a spontaneous abortion (Figure 1).

### Relative affordability/unaffordability of the treatment costs associated with induced vs spontaneous abortion

Tables 4 and 5, respectively, show the relative affordability of the costs associated with induced and spontaneous abortion and the proportion of households which resorted to coping strategies to pay health care costs. The average expenditure associated with abortion represented 15% of the GDP per capita for women with induced abortion and 9% of the GDP per capita for those with spontaneous abortion (Table 4). Additionally, 12% of the sample of women incurred subsequent economic consequences, as expressed by their need to resort to measures such as reducing expenses on essential needs or using their entire savings, etc., to pay for their hospitalization (Table 5). Compared with women with spontaneous abortion, women who had had an induced abortion seemed to have faced unaffordable abortions costs with a higher proportion of these women bearing the economic consequences associated with catastrophic health care payments (16% vs 11%). Moreover, the statistical difference between women with induced abortion and those with spontaneous abortion as to unaffordability of costs associated with abortions seems to suggest that access to abortion and/or postabortion care may have impoverished much more women with induced abortion compared with women with spontaneous abortion.

**Table 4:** Unaffordability of treatment cost relative to the type of abortion

	Average abortion cost in US$ (SD)	Average abortion cost in percentage of GDP per capita	Significance
			*P*-value
Induced abortion	89 (75)	15%	
Spontaneous abortion	56 (50)	9%	<0.001

**Table 5:** Households’ recourse to coping strategies to pay care costs by type of abortion

	Women with unaffordable cost^a^	Women with affordable cost	Significance
	*N* (%)	*N* (%)	*P*-value
Induced abortion	6 (16)	30 (84)	
Spontaneous abortion	32 (11)	237 (89)	
All respondents	38 (12)	267 (88)	0.42

^a^Including use of entire savings, borrowing with high interest rates, selling household goods and assets, farm products, animals, reducing expenses on essential needs.

## Discussion

In this study, we have analysed the costs and consequences of abortion to women and their households. The study brought to light a number of results that are worthy of further discussion. The study demonstrated that women who had an induced abortion were dissimilar from women with spontaneous abortion as regards socio-economic characteristics. In line with previous other studies (Bankole *et al.* 2008; Ibrahim and Onwudiegwu 2012), a higher proportion of women who had an induced abortion compared with women with spontaneous abortion were young, educated, single, with no living children and experiencing their first pregnancy in life. In the context of Burkina Faso, these findings may have important implications considering the incidence of abortion in the youth (Rossier *et al.* 2006; Sedgh *et al.* 2011). Recent studies have demonstrated that improving information about and availability of contraceptives may reduce unwanted pregnancies and abortions, particularly in adolescents (Prata *et al.* 2011; van den Brink *et al.* 2011). In Burkina Faso, school adolescents tended to lack adequate information and access on/to family planning services. The promotion of sexual and reproductive health education for pre-adolescents in schools, along with the expansion of out-reach clinic work for family planning methods may be highly beneficial in reducing unwanted pregnancies (Rijsdijk *et al.* 2011), and therefore abortions and other risky sexual behaviours (Kirby *et al.* 2006). Additionally, reducing financial barriers to family planning services, especially for poor women and teenagers, may also yield positive effects on unwanted pregnancies and induced abortion rates. In this respect, eliminating unnecessary costs or excessive laboratory tests by health service providers could have a positive impact on the demand for such services.

Payments associated with abortions, whether the loss of the pregnancy was intentional or not, seemed to incur high expenses to women and their households. Moreover, the total cost to women who had induced abortions appeared very high compared with women with spontaneous abortion. This difference may be explained by higher hospital expenditures for the treatment of abortion complications to women with induced abortion than women with spontaneous abortion, in addition to the high costs incurred in order to terminate the pregnancy. These findings were also corroborated by several other studies that reported higher costs to women with induced abortion than women with spontaneous abortion (Henshaw *et al.* 2008; Naghma e 2011). Furthermore, we found that payments associated with induced abortion were on average unaffordable and led to some economic consequences for households. This was indicated by the households’ use of entire savings, by borrowing with high interest rates and by various other coping strategies to face costs, which suggested that payments associated with abortions consumed significant resources.

The study also highlighted the difference in total costs associated with abortions relative to the socio-economic status of households. We believe that long delays in decision-making before resorting to hospitals, due to the lack of means of payments, particularly for poor women, may have led to higher expenditures for these women through a greater deterioration of their health. We also think that women’s lack of control over household resources may have contributed to delaying their recourse to postabortion care. This may have led to more severe complications and much higher costs for these women, compared with women from better-off households.

Women who had induced abortions seemed to have faced unaffordable costs compared with women with spontaneous abortion. Scientific literature strongly highlighted socio-economic disparities as an important determinant in the access to maternity and pregnancy care (Ronsmans *et al.* 2006). Poor women may be less able to afford skilled care along the road to hospital, which is largely considered as essential to maternal mortality reduction (OMS 2005). In this study however, it is unclear whether payments associated with abortions affect mostly poor people or whether they also affect the wealthier ones. The relatively small size of the sample made it difficult to address the issue and further research is clearly needed.

The national subsidy policy for normal deliveries and emergency obstetric care that has been applied since 2006 in Burkina Faso specified that women should only pay US$7 for postabortion care. However, this study has demonstrated that women spent a minimum of US$19 for treating complications of abortions, independently of the type of abortion. This could have implications regarding the fight against maternal mortality and morbidity, as persistent high costs demonstrate it, in spite of the recommended subsidy policy. We did not find any significant difference in total abortion costs between the health structures in which the study was conducted. This suggests that the cost estimates we presented here may be more representative of that of women requiring care in these health structures. Therefore, these estimates may not be generalizable to the entire population of women with abortions.

### Limitations

This study faces a number of limitations. In assessing the affordability of abortion costs we used GDP per capita, which is an individual rather than a household level measure of income. Using this proxy measure may have overestimated income among unemployed women and students and did not allow the assessment of the relative affordability of costs among income groups. Moreover, in computing the asset index score, we considered the same assets for semi-rural and rural areas. By doing so, our analysis may have underestimated the utility function of assets such as a cart or plough for rural people compared with semi-rurals. Further, in analyzing the data, we excluded transport and opportunity costs. Transport costs appeared to be a non-negligible cost item, as women may travel long distances to reach health facilities (Naghma e 2011). Opportunity costs, i.e. loss of earnings due to illness, may have also exacerbated the impact of illness on households’ poverty. By excluding these costs, our cost estimates may have underestimated the true costs associated with abortions, especially those borne by women who had induced abortions and for whom, several journeys for seeking care may have been carried out. This also may have biased the real proportion of households affected by healthcare payments. In addition, women were interviewed at discharge and because of this, our estimations may have underestimated the real cost of abortions to women and their households. We believe this because some women, particularly those who had induced abortions, may have developed further complications which would have led to further expenses. However, because the women were interviewed at discharge, the recall time was not long enough to account for such events.

Furthermore, several studies emphasized the issue of misclassification of abortion cases, and the resulting biases that may affect studies’ findings (Singh 2006; Bernabé-Ortiz *et al.* 2009; Rasch 2011). The results of this study may have also been affected by misclassification of abortion cases, which may have significant impacts on the findings. In this study in particular, an underestimation of induced abortion cases may have occurred, whereas for spontaneous abortion an overestimation of the cases may be observed. Understatement and overstatement of abortion cases may have led, in turn, to an underestimation or overestimation of spontaneous and induced abortion costs, depending on how the cost to women who were inaccurately classified will differ from the mean cost to women with genuinely spontaneous abortions. For example, if this cost is lower than the mean cost to women with spontaneous abortion, an underestimation of the spontaneous abortion cost may be observed whereas an overestimation of the cost to women with induced abortion may be observed. A reverse correlation may be observed if this cost is higher than the mean cost to women with spontaneous abortion. In consequence, the economic repercussions of the payments may have been inflated or deflated for the groups of women. Because of this, we think that the differences in costs between women with spontaneous and induced abortions may have been biased by classification errors. The slight difference in consequences that we observed between our abortion groups may also be a result of misclassification of some induced abortions into spontaneous abortions. Finally, this study may also have suffered from selection biases. Abortion being restricted in Burkina Faso, women with induced abortion may be reluctant to seek help and thus present to hospitals with more severe complications whereas most of the women with spontaneous abortion may enter hospitals with less severe complications. This may have also affected the findings of the study.

### Strengths

Despite the limitations we pointed out, this study has some strengths. Prospective collection of data within a 2-week period of discharge may have contributed to reducing the effects of recall bias, especially for the collection of expenditure data. Additionally, each individual questionnaire was rigorously monitored twice to ensure consistency in responses. Furthermore, in Africa, the existing literature on costs associated with abortions to women and their households comes from English speaking countries whereas this study was carried out in a francophone low resource setting in which cultural thinkings are pro-natalist and abortions are prevalent. Finally, this study is the first study ever to be published that explores the costs of abortions to households in Burkina Faso.

## Conclusion

This study explored the costs of induced and spontaneous abortions to women and their households. It emphasized the costs borne by households and the short-term economic repercussions of payments associated with abortions. The findings of this study highlighted the critical need for financial protection mechanisms able to both reduce costs associated with abortions and the economic repercussions on women and households. In Burkina Faso, the persistent hurdles that payments associated with obstetric and delivery care facing households have prompted the Government to introduce in 2006 a national subsidy policy for normal and emergency obstetric deliveries. Despite the policy clearly stipulating a tariff of US$7 (3600 CFA) for postabortion care, this study brought evidence that women continue to pay significantly more (at least US$19) than the official tariff. An important implication is that the normal and emergency deliveries subsidy alone is not sufficient in itself in significantly reducing financial barriers to care and to ensure more financial protection. We believe a monitoring of its implementation on a regular basis, may be highly beneficial both to ensure that the policy is rigorously applied and that the targeting mechanisms effectively reach the people who need it.

## References

[czu025-B1] Åhman E, Shah IH (2011). New estimates and trends regarding unsafe abortion mortality. International Journal of Gynecology and Obstetrics.

[czu025-B2] Babigumira JB, Stergachis A, Veenstra DL (2011). Estimating the costs of induced abortion in Uganda: a model-based analysis. BMC Public Health.

[czu025-B3] Banerjee SK, Andersen K (2012). Exploring the pathways of unsafe abortion in Madhya Pradesh, India. Global Public Health.

[czu025-B4] Bankole A, Sedgh G, Oye-Adeniran BA (2008). Abortion-seeking behaviour among Nigerian women. Journal of Biosocial Science.

[czu025-B5] Benson J, Andersen K, Samandari G (2011). Reductions in abortion-related mortality following policy reform: evidence from Romania, South Africa and Bangladesh. Reproductive Health.

[czu025-B6] Berer M (2004). National laws and unsafe abortion: the parameters of change. Reproductive Health Matters.

[czu025-B7] Bernabé-Ortiz A, White P, Carcamo C (2009). Clandestine induced abortion: prevalence, incidence and risk factors among women in a Latin American country. Canadian Medical Association Journal.

[czu025-B8] Garner P, Meremikwu M, Volmink J, Xu Q, Smith H (2004). Putting evidence into practice: how middle and low income countries “get it together”. British Medical Journal.

[czu025-B9] Griebel G, Halvorsen J, Golemon T, Day A (2005). Management of Spontaneous Abortion. American Family Physician.

[czu025-B10] Guttmacher Institute (2014). Abortion in Burkina Faso, Fact Sheet.

[czu025-B11] Henshaw SK, Adewole I, Singh S (2008). Severity and cost of unsafe abortion complications treated in Nigerian hospitals. International Family Planning Perspectives.

[czu025-B12] Hogan MC, Foreman KJ, Naghavi M (2010). Maternal mortality for 181 countries, 1980–2008: a systematic analysis of progress towards Millennium Development Goal 5. The Lancet.

[czu025-B13] Ibrahim IA, Onwudiegwu U (2012). Sociodemographic determinants of complicated unsafe abortions in a semi-urban Nigerian town: a four-year review. The West Indian Medical Journal.

[czu025-B14] Johnston HB, Gallo MF, Benson J (2007). Reducing the costs to health systems of unsafe abortion: a comparison of four strategies. The Journal of Family Planning and Reproductive Health Care.

[czu025-B15] Kirby D, Obasi A, Laris BA (2006). The effectiveness of sex education and HIV education interventions in schools in developing countries. World Health Organization Technical Report Series.

[czu025-B16] Ministère de la santé Burkina Faso (2006). Arrêté 2006-002/PRES/PM/MS portant liste des accouchements et des soins obstétricaux et néonataux d’urgence subventionnés et leurs tarifs dans les formations sanitaires publiques de l’Etat.

[czu025-B17] Ministère de la santé Burkina Faso (2010). Annuaires Statistiques 2009.

[czu025-B18] Naghma e R (2011). Cost of the treatment of complications of unsafe abortion in public hospitals. Journal of the Pakistan Medical Association.

[czu025-B19] Okonofua F (2006). Abortion and maternal mortality in the developing world. Journal of Obstetrics and Gynaecology Canada.

[czu025-B20] OMS (2005). Pour une grossesse à moindre risque: Le rôle capital de l’accoucheur qualifié: Une déclaration conjointe OMS, ICM, FIGO.

[czu025-B21] Prata N, Bell S, Holston M, Gerdts C, Melkamu Y (2011). Factors associated with choice of post-abortion contraception in Addis Ababa, Ethiopia. African Journal of Reproductive Health.

[czu025-B22] Rasch V (2011). Unsafe abortion and postabortion care—an overview. Acta Obstetricia et Gynecologica Scandinavica.

[czu025-B23] Ridde V, Meessen B, Kouanda S (2011). Selective free health care in sub-Saharan Africa: an opportunity for strengthening health systems?. Sante Publique.

[czu025-B24] Rijsdijk L, Bos A, Ruiter R (2011). The World Starts with Me: a multilevel evaluation of a comprehensive sex education programme targeting adolescents in Uganda. BMC Public Health.

[czu025-B25] Ronsmans C, Holtz S, Stanton C (2006). Socioeconomic differentials in caesarean rates in developing countries: a retrospective analysis. The Lancet.

[czu025-B26] Rossier C, Guiella G, Ouedraogo A, Thieba B (2006). Estimating clandestine abortion with the confidants method—results from Ouagadougou, Burkina Faso. Social Science & Medicine.

[czu025-B27] Russell S (2004). The economic burden of illness for households in developing countries: a review of studies focusing on malaria, tuberculosis, and human immunodeficiency virus/acquired immunodeficiency syndrome. The American Journal of Tropical Medicine and Hygiene.

[czu025-B28] Sedgh G, Rossier C, Kaboré I, Bankole A, Mikulich M (2011). Estimating abortion incidence in Burkina Faso using two methodologies. Studies in Family Planning.

[czu025-B29] Sedgh G, Singh S, Shah IH (2012). Induced abortion: incidence and trends worldwide from 1995 to 2008. The Lancet.

[czu025-B30] Shah IH, Åhman E (2012). Unsafe abortion differentials in 2008 by age and developing country region: high burden among young women. Reproductive Health Matters.

[czu025-B31] Shearer JC, Walker DG, Vlassoff M (2010). Costs of post-abortion care in low- and middle-income countries. International Journal of Gynecology and Obstetrics.

[czu025-B32] Singh S (2006). Hospital admissions resulting from unsafe abortion: estimates from 13 developing countries. The Lancet.

[czu025-B33] Singh S (2010). Global consequences of unsafe abortion. Womens Health (London, England).

[czu025-B34] Singh S, Garcia SG, Guillaume A, Okonofua F, Prata N (2012). The health, social, and economic consequences of unsafe abortion: papers presented at an IUSSP Seminar, Mexico, 2010. International Journal of Gynaecology and Obstetrics.

[czu025-B35] Starrs AM (2006). Safe motherhood initiative: 20 years and counting. The Lancet.

[czu025-B36] The World Bank (2014). World DataBank: World Development Indicators.

[czu025-B37] United Nations (UN) (2013). The Millennium Development Goals Report.

[czu025-B38] van den Brink MJ, Boersma AA, Meyboom-de Jong B, de Bruijn JG (2011). Attitude toward contraception and abortion among Curacao women. Ineffective contraception due to limited sexual education?. BMC Family Practice.

[czu025-B39] Vlassoff M, Fetters T, Kumbi S, Singh S (2012). The health system cost of postabortion care in Ethiopia. International Journal of Gynaecology and Obstetrics.

[czu025-B40] Vlassoff M, Shearer J, Walker D, Lucas H (2009a). Economic Impact of Unsafe Abortion-Related Morbidity and Mortality: Evidence and Estimation Challenges.

[czu025-B41] Vlassoff M, Sundaram A, Bankole A, Remez L, Belemsaga-Yugbare D (2011). Benefits of meeting women’s contraceptive needs in Burkina Faso. Issues Brief (Alan Guttmacher Institute).

[czu025-B42] Vlassoff M, Walker D, Shearer J, Newlands D, Singh S (2009b). Estimates of health care system costs of unsafe abortion in Africa and Latin America. International Perspectives on Sexual and Reproductive Health.

[czu025-B43] World Health Organization (WHO) (2011). Unsafe Abortion: Global and Regional Estimates of the Incidence of Unsafe Abortion and Associated Mortality in 2008.

[czu025-B44] WHO (2012a). Safe Abortion: Technical and Policy Guidance for Health Systems.

[czu025-B45] WHO (2012b). Trends in Maternal Mortality: 1990 to 2010. WHO, UNICEF, UNFPA and the World Bank Estimates.

